# Impact of Somatosensory Input Deficiency on Subjective Visual Vertical Perception in Children With Reading Disorders

**DOI:** 10.3389/fneur.2019.01044

**Published:** 2019-10-01

**Authors:** Nathalie Goulème, Richard Delorme, Philippe Villeneuve, Christophe-Loïc Gérard, Hugo Peyre, Maria Pia Bucci

**Affiliations:** ^1^UMR 1141 NeuroDiderot Inserm – Université de Paris, Robert Debré Hospital, Paris, France; ^2^Posture Lab, Paris, France; ^3^Child and Adolescent Psychiatry Department, Robert Debré Hospital, Paris, France; ^4^Human Genetics & Cognitive Function, Institut Pasteur, Paris, France; ^5^Université de Paris, Paris, France

**Keywords:** brain, dyslexia, children, multisensory integration, cognitive rehabilitation

## Abstract

**Purpose:** Preliminary evidence indicated that children with a reading disorder (RD) may have deviance in their ability to perform high demanding cognitive tasks, such as reading, depending on somatosensory inputs. Until now, only anecdotical reports suggested that improving somatosensory inputs may influence their ability to maintain a stable perception of the visual world despite continuous movements of our eyes, head, and body. Here, we investigated whether changes in upright perception, the subjective visual vertical (SVV), were modulated by somatosensory inputs in a group of children with RD.

**Method:** The SVV task was used under two distinct conditions, i.e., with or without somatosensory inputs from the foot. We enrolled a group of 20 children with reading disorders and 20 sex-, age-, IQ- matched children with neurotypical development.

**Results:** Responses to the SVV task were found to be significantly less accurate in children with RD than in children with neurotypical development (*p* < 0.001). In the latter, SVV response did not depend on somatosensory inputs from the foot. In contrast, in children with RD somatosensory inputs, either improved or worsen their SVV depending on the tilt direction (*p* < 0.01).

**Conclusion:** Our results suggested that SVV responses in children with RD could be related to an immaturity for heteromodal sensory integration, including somatosensory inputs.

## Highlights

This original research article brings evidence that:

- In children with neurotypical development, subjective visual vertical perception appeared independent from the somatosensory inputs either to the tilt direction.
- Subjective visual vertical perception was deviant in children with reading disorder.
- Somatosensory inputs had an effect on visual vertical perception in children with reading disorder.
- These results suggest an immaturity for heteromodal sensory integration in children with reading disorder.

## Introduction

Reading disorder (RD), also called dyslexia, is a brain-based type of learning disability that specifically impairs a person's ability to read. Individuals with RD typically read at levels significantly lower than expected despite having normal intelligence. Although, this disorder varies from person to person, common characteristics among people with RD are difficulties with phonological processing (the manipulation of sounds), spelling, and/or rapid visual-verbal responding. RD have estimated prevalence rates of at least 10 percent of any given population (1), which imposes an enormous burden on society by impacting a huge number of children and adults, with far-reaching consequences across life domains.

During the last decade, consisting studies report that individuals with RD display a deficit to the abstract visual representation of letters. This deficit has been associated with a hypoactivation of the ventral occipitotemporal cortex, a region that has been closely associated with reading through the extraction of a representation of words, which is invariant to position, size, font, or case (2). Individuals with RD also display a deficit in spatial body representation and localization, associated with an impairment in their own motion detection perception (3). This global distortion of self-spatial representation is also correlated with a deficiency in postural control (4). Most of these findings point toward an impairment in cerebellar integration of complex sensory inputs. The cerebellum receives a rich input from visual, auditory, proprioceptive, and motor magnocellular systems (5). A desynchrony of sensory feedback and motor outflow affects the cerebellum functions as the control for automatizing motor skills (6). For example, in one of our studies, we observed that the postural instability in children with RD was more pronounced if one decreased somatosensory input. When using foam under their feet, children with RD significantly worsen their postural instability, compared to children with neurotypical development (4). Children with RD showed less accurate compensatory sensory strategies to maintain an efficient postural stability, when further affecting the feedback of somatosensory inputs (7).

Few studies tried to compensate for the deficit in postural stability observed in children with RD by modulating selectively the sensory inputs. Quercia et al. (8), however, explored the impact of visual and somatosensory inputs on postural stability in children with RD by studying the impact of prism and foam on postural sway. They reported an improvement on postural control for children with RD having both prism and foam compared to those without prism and foam. These results suggest that the increase of the salience of sensory information may slightly improve the postural stability in patients with RD. By extension, it may also participate in the improvement of their reading disability as reported in a small number of dyslexic children by Quercia et al. (9).

Based on this presuppose, there are anecdotical reports or studies in which patients used prism to improve the encoding of visual information to influence their cognitive resources dedicated to reading. Additional studies are needed to settle evidence, suggesting a need for reinforcing sensory integration in children with RD to compensate for their deficit in reading.

To further understand the influence of sensory modulation on postural stability, we decided to further explore the subjective visual vertical (SVV) ability in children with RD. SVV is defined by the ability to perceive gravitational orientation i.e., to estimate verticality in relation to the earth, in the absence of any external reference frame. SVV is essential to maintain stability and achieve postural tasks efficiently in daily life, given that most of our motor actions are achieved around the vertical axis (10). SVV has a role in our ability to maintain a stable perception of letters, despite continuous movements of our eyes, head, and body. However, some interesting studies by Harris's group reported that other techniques, such as perceptual upright and/or the subjective haptic vertical (SHV), can give more precise information of subjective perceived direction of gravity [see (11–13)]. Interestingly, the tasks developed to explore SVV allow the investigation of spatial cognitions, vestibular functions and verticality functions, involved in postural control (14). The SVV ability relies on a specific and large brain cortical network (15)—involving the right lateral temporo-occipital cortex, and the bilateral temporo-occipital and parieto- occipital cortical areas—but also the cerebellum (16).

Our present study compared the SVV in a group of 20 children with RD and in a group of 20 sex-, age-, and IQ- matched children with typical neurodevelopment, under two distinct conditions depending on somatosensory inputs (using foam under their feet). We hypothesized that children with RD displayed a more severe SVV than children with typical neurodevelopment, and that this impairment is worsened (in both groups but deeper in children with RD) when interfering with the somatosensory information, i.e., by decreasing the foot somatosensory inputs with the foam.

## Methods

### Participants

Twenty children (4 girls and 16 boys) with RD, and 20 sex-, age-, and IQ- matched children with neurotypical development participated in the study (see Table 1). Anova run on age means failed to show a significant difference between the two groups [*F*_(1, 38)_ = 0.01, *p* = 0.90] Children with RD were recruited at the Child and Adolescent Psychiatry Department (Robert Debré Hospital, Paris, France) to which they were referred for a throughout neuropsychological and phonological exploration. To be included, children should not receive any psychotropic drug at time of the study, should not display any personal history of somatosensory abnormalities, should have a normal visual acuity at both eyes and should have an IQ in the normal range (85–115) based on the Weschler's scale 4th edition (mean full scale IQ was 103 ± 1.1). Control individuals were children from the general population with no personal history of atypical neurodevelopment. Their cognitive abilities were estimated using two subtests of the Weschler's scale 4th edition, i.e., the similarities subtest which was used as an estimation of the verbal IQ and the matrix reasoning subtest which correlated with the non-verbal IQ. There was no significant difference in the mean scores of the two subtests when comparing children with RD and controls (12 ± 1.0 vs. 12 ± 1.5 and 11 ± 1.0 vs. 10.8 ± 2.0, similitudes and matrix reasoning subtests, children with RD vs. children with neurotypical development, respectively).

**Table 1:** Age (mean and standard error) and sex of the two groups of children tested.

	**Age (years)**	**Sex**
Children with ND	9.43 ± 0.22	4 girls, 16 boys
Children with RD	9.39 ± 0.37	4 girls, 16 boys

For each child with RD, enrolled in the study, the time required to read a text was assessed, as well as the general text comprehension and the ability to read words and pseudo-words, using the L2MA battery (17). This phonologic battery developed 30 years ago was still considered as the gold standard instrument in France to diagnose a RD in children. Children with a mean score beyond 1.5 standard deviation at the L2MA battery were diagnosed with RD.

The investigation adhered to the principles of the Declaration of Helsinki and was approved by our Institutional Human Experimentation Committee. Written informed consent was obtained from the children's parents after an explanation of the experimental procedure was provided.

### Subjective Visual Vertical Task

A wall case projects a laser stripe, with a precision <0.5° and a resolution <0.1°. This laser strip was projected on a blank wall, in a dark room distant 2.50 m from the child. This laser stripe can be rotated silently by 15° toward the right (clockwise) and likewise toward the left (counterclockwise) with a remote control. The remote to adjust the orientation of this laser stripe could be used step by step or in continuous movement.

### Experimental Paradigm

Children had to stand up on their feet on the footprints of the multitest-equilibrium framiral platform with their arms alongside their body (FRAMIRAL, see for details www.framiral.fr). The position of their feet was on the footprints, heels distant of 2 cm and feet spread out in a symmetric way with respect to the sagittal axis of the child at a 30° angle. Instructions were given to each child in order to perform the SVV test properly. Each child had to inform the experimenter when he/she saw the laser stripe perfectly vertical. At the beginning of the experiment, the laser stripe projected on the wall, in front of the child, was perfectly vertical; then the experimenter inclined the laser strip in clockwise (+15°) or counterclockwise (−15°), randomly. The arrow moved continuously in one direction only. Unfortunately, this system did not allow to record the time answer.

**Figure 1:**
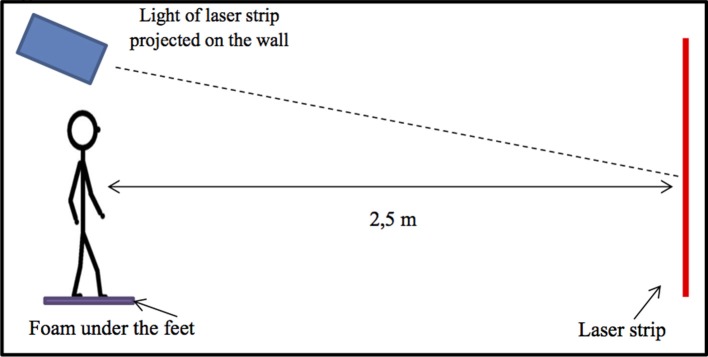
Experimental set up used. The child is standing on with the feet on foam (Orthomic®), and the laser strip is projected on the wall in front of him/her. The laser strip can be tilted at 15° in clockwise (CW) and in counterclockwise (CCW) directions, and the child has to inform the experimenter when he/she perceived the laser strip vertical. The remote to the orientation of the laser strip is quiet and can be used step by step or in continuous movement.

The child had to say when he/she saw the laser strip perfectly vertical according to him. Simultaneously, on a computer, the experimenter could read the tilt (in degrees) of the laser stripe projected on the wall. During the test, children wore special glasses, without any correction, only to retain the visual field and to avoid visual distractors. Indeed, children with RD were known to have a deficit in visual attention (18, 19). Thus, using these glasses allowed the child to focus only on the laser stripe projected in front of him/her. To evaluate the influence of somatosensory inputs from the foot on SVV responses, we used two different postural conditions i.e., with and without Orthomic® foam of 4 mm (with a density of 250 and a shore of 40) (Figure 1). These two postural conditions were randomly tested.

Five trials in each direction (clockwise or counterclockwise) were run randomly for each postural condition (with and without foam) to increase reproducibility and avoid risk of data heterogeneity.

### Data Analysis

We measured the SVV absolute values that were in degrees, the errors from the absolute visual vertical at zero degree. For each child, we calculated the average of the five trials performed in each direction (clockwise or counterclockwise) and for each postural condition (with and without foam).

### Statistical Analysis

Analysis of variance (ANOVA) was performed on mean SVV absolute values, considering the two somatosensory conditions i.e., with or without foam, and the two tilt conditions (clockwise and counterclockwise). *Post hoc* analysis was made with the Fisher's Least Significant Difference (LSD) test. A two-tailed *p* < 0.05 was considered statistically significant. All statistical analyses were conducted using SAS statistical software (version 9.4, Cary, North Carolina).

## Results

**Figure 2:**
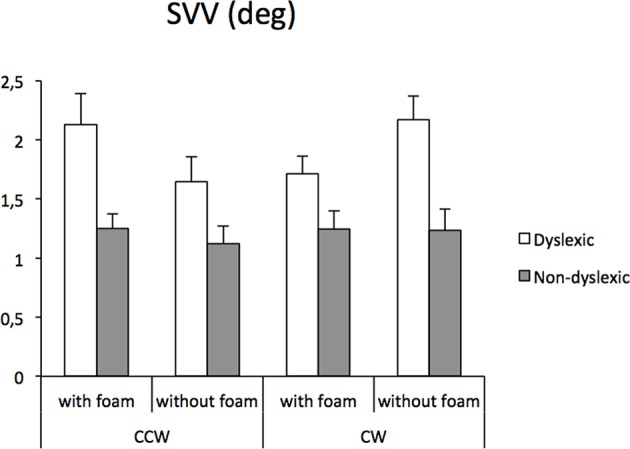
Mean subjective visual vertical and standard error measures (in degree) in children with reading disorder and children with neurotypical development, under two distinct somatosensory conditions (without and with foam under the feet) performed in each tilt direction [clockwise (CW) or counterclockwise (CCW)].

The SVV absolute measures in children with RD and in the sex-, age-, and IQ- matched children with neurotypical development were compared under the two distinct somatosensory conditions (without and with foam under the feet) when performing counterclockwise or clockwise tilt movements (Figure 2).

The ANOVA showed a significant group effect [*F*_(1, 38)_ = 22.31, *p* < 0.0001]; children with RD were significantly less accurate when processing SVV than children with neurotypical development. Whatever the conditions explored in the experiment, subjects with RD showed a severe deficit in SVV, reaching up to 50–80% of additional distortion compared to controls.

The ANOVA also reported a statistically significant interaction between groups, tilt condition, and the somatosensory information (the presence or the absence of the foam) [*F*_(1, 38)_ = 5.83, *p* < 0.02]. We observed in controls that the SVV was not affected by the somatosensory information whatever the tilt condition. There was no difference in the counterclockwise condition or in the clockwise condition. At the opposite, in the subjects with RD, we observed a significant difference depending on the modulation of the somatosensory input (with or without the foam), but with a divergent effect in the counterclockwise or in clockwise conditions (both *p* < 0.01). However, whatever the tilt conditions, SVV perception impairment remained significantly affected when compared to controls.

## Discussion

In our exploratory study, subjects with RD showed a severe deficit in SVV, reaching up to 50–80% of additional distortion, compared to controls. Indeed, children with RD displayed poor SVV perception, where somatosensory inputs either improved or worsened their SVV depending on the tilt direction.

Note that the direction effect was unexpected; in contrast, in line with our initial hypothesis, we reported that SVV in patients with RD were sensitive to somatosensory information, further stressing a potential impairment in cerebellar integration of complex sensory inputs in RD.

The SVV response in RD children depends on the tilt direction and on presence or absence of the foam. Indeed, the SVV perception for RD children without foam was significantly better in the CCW direction than in the CW direction, while with the foam under the feet, the results were the opposite: that is, the SVV perception was significantly better in the CW direction with respect to CCW direction. This could be due to the presence of attentional bias in RD population, as already reported by Michel et al. (20) and Vieira et al. (21), leading to such better performance in SVV measure when the vertical line is tilted on CCW direction. Indeed, Vieira et al. (21) described, in RD children, a proprioceptive bias in circle centering tasks, depending on hand position starting, while in age matched healthy children, neither asymmetry nor bias has been reported. Similarly to this proprioceptive bias, foam under the feet could impact SVV process.

Thus, somesthesic inputs from the foot sole influence the perception of SVV in RD children, most likely due to their difficulty to compensate somesthesic inputs, which are made misleading by foam. According to several studies, the thin thickness of such foam did not involve any change in motor activity but only involved a misleading of somesthesic input of sole foot, which can increase balance disorders (22–25). We suggest that clinicians need to pay attention to the use of foot sole in the dyslexic population. Further studies exploring the effect of foam on a large dyslexic population are required.

The estimation of SVV involves the allocentric, gravitational, and egocentric references, which are built by visual, vestibular, and somatosensory afferents (10, 26). The representation of self and environment around self is linked to these distinct sensorial modalities and requests a precise integration of complex sensory inputs (27). The brain structures which are involved in SVV modulation have been well-described, stressing the role of cortical and subcortical structures, mainly the temporo-occipital, parieto-temporal, parieto-occipital, and insular cortex but also the posterior-lateral thalamus (28, 29). SVV also involves the vestibular areas and the cerebellum (16). Part of this multimodal network also controlled the postural stability (specifically the cerebellum), which could explain why we previously found a postural instability in children with RD (4). Additionally, earlier findings reported that the ability for gravitational reference, as well as self-representation in space around the vertical axis, depends on postural stability (30, 31).

The role of the cerebellum is probably the corner piece in the SSV impairment that we reported, by its role in the treatment of complex cognitive skills and in the integration of sensory inputs. The difficulties in accurate and fluent written word recognition reported in patients with RSD was also associated with a cerebellum dysfunction (32, 33). In a meta-analysis of four functional and structural imaging studies in children (5–15 years of age), a reduction in gray matter volume in both hemispheres of the cerebellum were observed in individuals with RD (34). Similarly, results were reported in large-scale studies in adults with RD showing a decreased local gray matter volume in the right cerebellum and in the right lentiform nucleus (35) or in the left posterior cerebellum (36).

The potential cerebellar dysfunction in RD may explain why the modulation of somatosensory inputs had an impact on SVV in our study. We observed that children with RD displayed more difficulties for fine-grain control of the SVV in the context of somatosensory input deficit (i.e., in the presence of the foot foam). This effect does not seem related to a change in motor stability, as consistently reported by previous findings (24), but only to a misleading of somatosensory input of ground.

For children with neurotypical development, we did not observe any effect of the modulation of the somatosensory input, suggesting that compensatory mechanisms involved in SVV response were efficient. In one of our previous studies, we also observed that SVV response was independent from foot sensory inputs, whatever the tilt directions in healthy children (37). Similar results were obtained when exploring the effect of foam on postural control in children with neurotypical development (4). This further suggested that perturbation of ground inputs were counteracted by additional sensory afferences to ensure an optimal postural stability (16).

## Conclusion

To conclude, this current study showed that children with RD display a deficit in SVV process compared to children with neurotypical development. The use of foam as a proxy of sensory input privation affected the SVV perception only in children with RD, suggesting poor compensatory mechanisms in the treatment of information, ensuring an appropriated representation of self and of the environment in space. The role of sensory inputs has to be further considered in children with RD, and could shed new perspectives in treatment management and cognitive rehabilitation of these patients.

## Limitations

Note that SVV evaluation used in this study has some limitations; for instance, it was not possible to control for other confounding variables that effect the response time of children. Further studies exploring the effect of other types of somatosensory inputs in RD children through using other experimental techniques, such as the Oriented CHAracter Recognition Test (OCHART) from Harris's group (38), will be useful to better understand the impairment of such inputs in visual vertical perception performance in children with RD, allowing for the development of specific rehabilitation. Finally, one could ask the question whether postural sway could bias the SVV perception, given that it is well-known that children with RD have poor postural stability (7). Further studies recording simultaneously postural sway and SVV perception in children with RD are needed in order to further explore their mutual interaction.

## Data Availability Statement

Publicly available datasets were analyzed in this study. This data can be found here: data are in the text.

## Ethics Statement

The studies involving human participants were reviewed and approved by the investigation adhered to the principles of the Declaration of Helsinki and was approved by our Institutional Human Experimentation Committee. Written informed consent was obtained from the children's parents after an explanation of the experimental procedure was provided. Written informed consent to participate in this study was provided by the participants' legal guardian/next of kin.

## Author Contributions

NG, PV, and MB: conceptualization. C-LG and HP: selection of patients. NG and MB: SVV measure and data analysis. NG, RD, HP, and MB: writing original draft. PV and C-LG: review and editing. All persons designated as authors qualify for authorship. Each author participated sufficiently in the work to take public responsibility for appropriate portions of the content.

### Conflict of Interest

The authors declare that the research was conducted in the absence of any commercial or financial relationships that could be construed as a potential conflict of interest.
